# The Role of Histidine-Proline-Rich Glycoprotein as Zinc Chaperone for Skeletal Muscle AMP Deaminase

**DOI:** 10.3390/biom4020474

**Published:** 2014-05-05

**Authors:** Maria Ranieri-Raggi, Arthur J. G. Moir, Antonio Raggi

**Affiliations:** 1 Laboratory of Biochemistry, Department of Pathology, University of Pisa, via Roma 55, Pisa 56126, Italy; E-Mail: maria.ranieri@med.unipi.it; 2 Department of Molecular Biology and Biotechnology, Krebs Institute, University of Sheffield, Sheffield S10 2UH, UK; E-Mail: a.j.moir@sheffield.ac.uk

**Keywords:** AMP deaminase, histidine-proline-rich glycoprotein, zinc chaperone, zinc binding site

## Abstract

Metallochaperones function as intracellular shuttles for metal ions. At present, no evidence for the existence of any eukaryotic zinc-chaperone has been provided although metallochaperones could be critical for the physiological functions of Zn^2+^ metalloenzymes. We propose that the complex formed in skeletal muscle by the Zn^2+^ metalloenzyme AMP deaminase (AMPD) and the metal binding protein histidine-proline-rich glycoprotein (HPRG) acts in this manner. HPRG is a major plasma protein. Recent investigations have reported that skeletal muscle cells do not synthesize HPRG but instead actively internalize plasma HPRG. X-ray absorption spectroscopy (XAS) performed on fresh preparations of rabbit skeletal muscle AMPD provided evidence for a dinuclear zinc site in the enzyme compatible with a (μ-aqua)(μ-carboxylato)dizinc(II) core with two histidine residues at each metal site. XAS on HPRG isolated from the AMPD complex showed that zinc is bound to the protein in a dinuclear cluster where each Zn^2+^ ion is coordinated by three histidine and one heavier ligand, likely sulfur from cysteine. We describe the existence in mammalian HPRG of a specific zinc binding site distinct from the His-Pro-rich region. The participation of HPRG in the assembly and maintenance of skeletal muscle AMPD by acting as a zinc chaperone is also demonstrated.

## 1. Introduction

Transition metal ions play an essential role in many major processes in biochemistry. Cells employ transition metal ions in structurally constrained binding sites in metalloproteins, where they can carry out structural, regulatory or catalytic roles. Metalloenzymes employ transition metals as co-factors. The half life for dissociation of metal ions from metalloenzymes is days to weeks. Thus, many metalloenzymes retain their cofactor throughout the lifetime of the cell [1]. Since these metal ions can also catalyse cytotoxic reactions, several families of proteins are present in cells that regulate and determine the concentration of these transition metals and confine them to vital roles [2]. An emerging new family of soluble metal receptor proteins, metallochaperones, acts in the intracellular trafficking of metal ions. These metal receptors function as intracellular shuttles for metal ions, acquiring the metal ion and delivering it to specific partner proteins [3]. Zinc is an essential metal ion and is the second most abundant transition metal ion in living organisms after iron. Cells stringently regulate their intracellular zinc levels, since too little zinc inhibits metabolism and high concentrations of zinc are toxic to cellular functions [2]. Zinc homeostasis in eukaryotic cells is controlled by several types of proteins that influence the levels of uptake (plasma membrane zinc importers), intracellular sequestration in zinc storing vescicles (zincosomes), nucleocytoplasmic distribution (metallothionein) and elimination (zinc exporter proteins) [4]. Many cellular processes such as signal transduction, cell proliferation and differentiation involve zinc. At the molecular level, zinc is a structural constituent of a great number of proteins, including enzymes and transcription factors [4]. At the present time, the only eukaryotic metallochaperones that have been identified are copper-chaperones [3]. No evidence for the presence of any zinc-chaperone has been provided, although in certain situations it has been suggested [5] that metallothioneins may be considered to be zinc chaperones. However, it has been reported that the total cytoplasmic free zinc concentration is very low (about 10^−10^ M) similar to that determined in plasma, even in tissues where the total Zn^2+^ is unusually high (e.g., 1 mM in muscle) [6]. This observation strongly suggests the presence of intracellular Zn-chaperones acting to make free zinc available for partner proteins could be critical for the physiological functions of Zn^2+^ metalloenzymes. This is particularly true for skeletal muscle taking into account the results of the recent studies on the novel protein-protein complex formed by the Zn^2+^ metalloenzyme AMP deaminase (AMPD, EC 3.5.4.6) and the zinc binding protein histidine-proline-rich glycoprotein (HPRG) [7,8,9,10,11].

AMPD is a multimeric enzyme that was first isolated by Schmidt in 1928 [12]. Extensive work carried out by Parnas and his school well established that ammonia formed by muscle during work arises from deamination of AMP to IMP catalyzed by AMPD [13,14]. Parnas considered irreversible the deamination processing of AMP, which could occur during anaerobic contraction, and hypothesized the existence of a regenerative process of AMP from IMP occurring during periods of oxidative recovery [15]. The results of subsequent work have fully confirmed the hypothesis presented by Parnas. Lowenstein and Tornheim [16] gave evidence for the occurrence in muscle of a cyclical process, termed the purine nucleotide cycle, consisting of the reactions catalyzed by AMPD, adenylosuccinate synthetase and adenylosuccinase. Although the precise physiological role of this cycle is not known, there is no doubt that the removal of AMP by deamination plays a significant function in the regulation of the energy charge, *i.e.*, the relative concentrations of the adenine nucleotides AMP, ADP and ATP [17]. When utilization of ATP is kept low, the stabilization of the energy charge value is assured by the equilibrium of the adenylate kinase reaction (2ADP = ATP + AMP). Under conditions of impaired energy metabolism, such as during the sustained contractile activity that occurs with a rapid utilization of ATP, recovery of normal values of energy charge may be achieved by favouring the adenylate kinase reaction towards ATP production through the acceleration of the breakdown of AMP.

AMPD is widely distributed in animal tissues; however, its level of activity in skeletal muscle is particularly high when compared with that found in all other tissues, including heart and smooth muscle [18]. Enzyme distribution data showed that white muscles from different species deaminate AMP four to 10 times more effectively than red muscles that showed about 15% of the adenylate kinase activity of white muscle [19]. It has also been shown that AMPD exists in striated muscle as two different isoenzymes [20,21,22]. In the red muscles, the prevalent form has the chromatographic properties of the single form present in the heart, while the minor form of AMPD found in red muscles corresponds to that which accounts for the greatly increased AMPD activity of white muscles [20]. Since the rabbit skeletal muscle apparatus is generally composed of white muscle, rabbit skeletal muscle AMPD preparations show the considerably higher specific activity of the isoform specific to white muscle (isoform M) that shows a peculiar inhibition by ATP, especially at pH values higher than neutrality, ADP being the most efficient metabolite in counteracting that inhibition [23].

HPRG is a single chain glycoprotein that is present at a relatively high concentration in the plasma of vertebrates (100–150 µg/mL in humans) [24,25]. The physiological role of HPRG is still to be established, although it has been implicated in a number of cellular processes including blood coagulation and fibrinolysis, angiogenesis, immune complex clearance, cell adhesion and cell migration [26]. HPRG can bind to a variety of ligands such as fibrinogen, plasminogen, heparin, heparan sulphate, tropomyosin, heme and divalent transition metal cations such as zinc, copper, mercury, cadmium and nickel. It interacts with Zn^2+^ with a stoichiometry of approximately 1:1–1:10 [24]. The interaction between HPRG and Zn^2+^ is abolished when histidine residues are chemically removed from HPRG, supporting the model that histidine residues are essential for Zn^2+^ binding [27].

It has been shown that a HPRG-like molecule is associated to purified rabbit skeletal muscle AMPD [7] and that the separation by zinc-affinity chromatography of the HPRG-like component markedly reduces the solubility of the catalytic subunit of the enzyme [9], strongly suggesting a role of HPRG in the maintenance of the native quaternary structure of the enzyme that could be envisaged from the formation of a 1:1 HPRG-AMPD molecular adduct [10].

## 2. General Aspects of the Molecular Structure of Rabbit Skeletal Muscle AMP Deaminase

A tetrameric structure has been suggested for skeletal-muscle AMPD from various species. However, a range of molecular masses (from 238–326 kDa) has been reported for the enzyme [28,29,30,31,32,33,34,35,36,37]. In the light of the observation that the rabbit enzyme undergoes fragmentation on storage, the *N*-terminal domain (10 kDa) being removed [38], an effect that can be reproduced by limited trypsinization which cleaves the enzyme subunit (85 kDa) after lysine-95 [39,40], the different reports on the molecular mass of skeletal-muscle AMPD have been interpreted as being due to the inherent instability of the enzyme during the prolonged extraction step that is performed at room temperature.

The determination by sedimentation-equilibrium analysis of the molecular mass of freshly prepared rabbit skeletal muscle AMPD indicated the presence of two species of 173 kDa and 309 kDa, which were interpreted as being consistent with the existence of a dimer-tetramer equilibrium [41]. It was also shown that after limited proteolysis with trypsin, rabbit skeletal muscle AMPD sediments as a single species of 222 kDa [41]. At that time, this observation was taken as suggestive of limited proteolysis “freezing” the enzyme in a trimeric conformation state. In the light of the subsequent observation of the association of rabbit skeletal muscle AMPD with a HPRG-like molecule [7], the 309 kDa molecular mass previously determined for the native enzyme is in total agreement with a new model for AMPD, a 1:1 molecular adduct in which two 85 kDa catalytic subunits assemble with two HPRG subunits (approx. 70 kDa each); furthermore, the 222 kDa molecular mass determined for the trypsinised enzyme can be explained by taking into account the observation that limited proteolysis of AMPD by trypsin also liberates from the HPRG component a 30 kDa fragment [7], thereby suggesting a tetramer assembly for proteolysed AMPD formed by two *N*-truncated catalytic subunits of approximately 75 kDa and two proteolysed HPRG subunits of approximately 40 kDa [10].

Analysis of the chromatographic behaviour of recombinant wild-type AMPD1 has given very different results since the protein appeared to aggregate into a large complex (>2000 kDa) [42]. This observation may well be due to the instability of the isolated native 85 kDa catalytic subunit resulting in an aggregation-induced precipitation, further emphasising the association with the HPRG component as being critical for the correct assembly of a stable AMPD complex. This view is strengthened by the kinetic properties of the recombinant enzyme [42] that differ sharply from those established in the literature for native skeletal muscle AMPD (e.g., eight times higher Km, strong activation by ATP).

No crystal structures are presently available for the native form of AMPD. From the literature, the only available AMPD crystal structures are those of rabbit AMPD1 and of its plant ortholog encoded by Arabidopsis embryonic factor 1 FAC1) [43,44]. It should be noted, however that in both cases, crystallisation was performed using *N*-truncated AMPDs to overcome the problems deriving from the instability of the native proteins. In particular, AMPD1 crystals were prepared from frozen rabbit muscle, although it is widely accepted that the rabbit enzyme undergoes fragmentation on storage. In contrast to the fragmented species that is isolated from frozen tissue, a very stable AMPD preparation can be obtained from fresh rabbit skeletal muscle, the 85–70 kDa SDS gel band transition of the purified enzyme occurring with a half-time of one month [10]. X-ray crystallography of the two *N*-truncated enzymes suggest that the oligomeric make-up of plant and animal AMPD are quite different, since a tetramer is observed in the crystals of rabbit AMPD1, whilst FAC1 assembles as a physiological dimer. Notably, the FAC1 dimer has a larger contact area than any two rabbit AMPD1 subunit contacts. These discordant observations strongly suggest that the quaternary structure of a biochemically significant form of skeletal muscle AMPD can be unambiguously established only when the structure determination is undertaken on the native whole enzyme, isolated from fresh tissue.

## 3. Rabbit Skeletal Muscle AMP Deaminase Is a Metalloenzyme with a Dinuclear Zinc Site

The characterization of skeletal muscle AMPD as a zinc metalloenzyme was reported for the rat enzyme [45] as well as for the rabbit enzyme [32] on the basis of its interaction with chelating agents and metal ions. Atomic absorption analysis established the presence of 2.0 and 2.6 g atoms of zinc, respectively, per mole of rat enzyme (mol wt 290 kDa) and rabbit enzyme (mol wt 278 kDa) [32,45]. The problem of assigning a precise stoichiometry for the zinc binding to rabbit skeletal muscle AMPD is complicated by the observation that the apoenzyme binds 4 g atoms of zinc per mol, but the increase of Vmax due to the addition of the fourth zinc atom is only 28% of that expected. This suggests that the fourth zinc atom is not directly associated with activity [32].

More recently, one zinc atom content was reported for the 80 kDa subunit of a form of AMPD from baker’s yeast that lacks an *N*-terminal segment of 192 amino acids as a consequence of a proteolytic cleavage that occurs during purification [46]. Alignment of the amino acid sequence for yeast AMPD with that for mouse adenosine deaminase demonstrated conservation of the four amino acid residues (3 His and 1 Asp) known from the X-ray crystal structure of adenosine deaminase to bind zinc in contact with the attacking water nucleophile [47]. On the basis of these similarities, the same model of a penta-coordinated zinc bound at the catalytic site that was described for adenosine deaminase, a 352 amino acids protein, has also been proposed for the 810 amino acid monomer of yeast AMPD [46].

Comparison of the sequences of rat and human skeletal muscle AMPD [48] with the sequence predicted from a genomic sequence of rabbit AMPD1 (isoform M) (accession XP_002715794) shows a very high degree of conservation of sequences in the *C*-terminal regions involved in zinc binding, but alignment of the amino acid residues supposed to be in contact with zinc in yeast AMPD [46] with the deduced amino acid sequence for the skeletal muscle enzyme demonstrates conservation of only three amino acid residues (His-363, His-572 and Asp-649, corresponding respectively to His-422, His-630 and Asp-707 in yeast AMPD), whilst His-424 of the yeast enzyme is replaced by Gly-365 in the skeletal muscle enzyme of the mammalian species.

**Figure 1:**
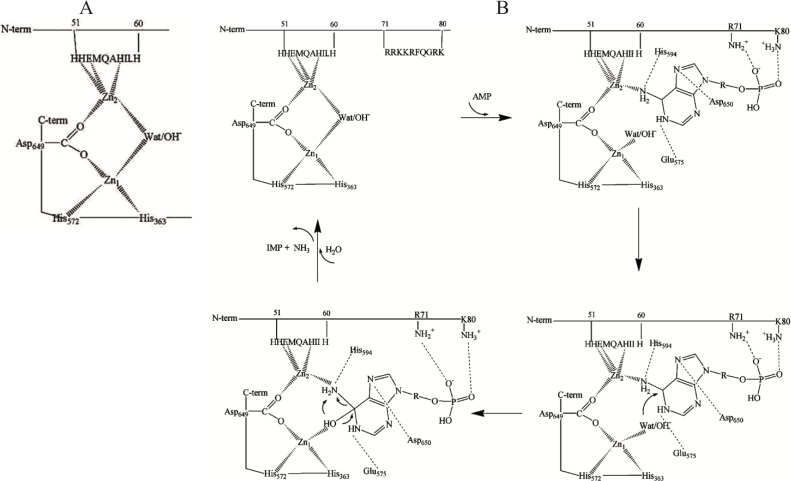
(**A**) A model of dinuclear Zn site in rabbit skeletal muscle AMP deaminase (AMPD) [49]; (**B**) Proposed interactions of the substrate at the dinuclear cocatalytic Zn site of rabbit skeletal muscle AMPD [50]. The amino acid residues involved in the binding in the catalytic Zn_1_ region are based on the proposed interactions of the substrate transition state at the catalytic site of adenosine deaminase [47] and amino acid homology to rabbit AMPD1.

Taken together, these observations confirm that zinc is a firmly bound component of skeletal muscle AMPD and is essential for enzyme activity but also suggest that the model of the zinc binding site proposed for yeast AMPD cannot be unambiguously extended to the skeletal muscle enzyme. Moreover, X-ray absorption spectroscopy of Zn-peptide binary and ternary complexes prepared using a number of synthetic peptides mimicking the potential metal binding sites of rabbit skeletal muscle AMPD strongly suggested that the region 48–61 of the enzyme contains a zinc binding site whilst region 360–372 is not able to form 1:1 complexes with zinc, in contrast with what has been suggested for the corresponding region of yeast AMPD [49]. X-ray absorption spectroscopy performed on fresh preparations of rabbit skeletal muscle AMPD provided evidence for a dinuclear zinc site in the enzyme compatible with a (µ-aqua)(µ-carboxylato)dizinc(II) core with an average of two histidine residues at each metal site and a Zn–Zn distance of about 3.3 Å [50]. The data also indicated that two zinc ions are bound to the catalytic subunit of the enzyme, one to the three conserved amino acid residues among those four assumed to be in contact with zinc in yeast AMPD, and the other at the *N*-terminal region, probably to His-51, Glu-53 and His-57 (Figure 1A). Justification for a structurally bridged dinuclear metallocenter in rabbit skeletal muscle AMPD was further supported by *N*-terminal analysis of the peptides liberated by limited tryptic digestion of different enzyme preparations suggesting the existence of two different protein conformations which is consistent with the hypothesis of the presence of a zinc ion connecting the *N*-terminal and *C*-terminal regions of AMPD [49]. When the limited proteolysis affects only the *N*-terminal 1–95 residue region of the enzyme, the consequence on the constitution of the enzyme metallocenter is probably the loss of the putative Zn_2_ ion without any major change in the conformation of the remaining part of the enzyme. In contrast, when the digestion affects the binding region of the Zn_1_ ion at the *C*-terminus, profound changes within the AMPD structure ensue as suggested by the observed fragmentation in the middle- and *C*-terminal regions of the enzyme, whilst in contrast, the 58–95 region appears to be unaffected [49]. The hypothesis of a conformational change of AMPD induced or stabilized by zinc binding to the 51–60-residue region (HHEMQAHILH) is strengthened by the observation that carbethoxylation by diethyl pyrocarbonate of one or two histidine residues per subunit results in conversion of rabbit skeletal muscle AMPD into a species that, unlike the native enzyme, is not sensitive to regulation by ATP at optimal pH 6.5 [51].

The two Zn ions in the AMPD metallocenter operate together as a catalytic unit, but play different roles in the catalytic mechanism, one of them (Zn_1_) acting to polarize the nucleophile water molecule, whilst the other (Zn_2_), possibly in co-operation with Zn_1_, could act transiently as the receptor for an activating substrate molecule (Figure 1B). The ligand displacement that presumably occurs when substrate enters the zinc coordination sphere of AMPD is consistent with a *cocatalytic* mechanism [52].

In contrast with the isoforms from all other sources (including cardiac AMPD) that are activated by ATP, skeletal muscle AMPD shows a peculiar inhibitory effect by ATP [22,23]. Since ADP was shown to be the most efficient metabolite in counteracting the inhibition by ATP, a decrease in the ATP/ADP ratio, together with a decrease in the tissue pH, has been suggested to be the stimulus for the activation of AMPD in periods of intense muscular activity [23]. In the AMPD specific to rabbit fast-twitch muscle fibers, the putative Zn_2_ binding site might represent the regulatory site at which the competition between activatory and inhibitory adenine nucleotides could take place. This peculiar kinetic property of rabbit skeletal muscle AMPD is likely to be due to the operation of a regulatory anion-binding site consisting of a cluster of positive charges localised between residues 72 and 80 (RKKRFQGRK, Figure 1B) which could direct the binding of adenine nucleotides to the Zn_2_ ion binding region. Mild modification of rabbit skeletal muscle AMPD by trinitrobenzene sulfonic acid resulted in conversion of the enzyme into a species with about six trinitrophenylated lysine residues per molecule (mol wt 310 kDa) which no longer manifests positive homotropic cooperativity behaviour at pH 7.1 or at the optimal pH value of 6.5 in the presence of low K^+^ concentrations [53]. A near identity exists between the effects on the modulation of the enzyme by substrate brought about by lysine trinitrophenylation and those following the removal of the 95-residue long *N*-terminus of the enzyme by limited proteolysis with trypsin [39,41], emphasising that the *N*-terminal region of rabbit skeletal muscle AMPD encompasses that regulatory anion binding region. It was previously suggested that a proteinase present in muscle, as well as trypsin, could remove from AMPD a fragment acting at least partially to hold the enzyme in an inactive conformation [38] and, therefore, this phenomenon could be envisaged to be the basis for the large ammonia accumulation that occurs in skeletal muscle subjected to strong tetanic contraction or passing into *rigor mortis* [54]. One role of the region of AMPD that is removed by limited proteolysis could be the maintenance of the three-dimensional structure of the AMPD subunit in a specific conformation that causes the enzyme in the absence of activators to show homotropic positive cooperativity even at optimal acidic pH. It has also been suggested that the cleavage of rabbit skeletal muscle AMPD on storage is produced by a calpain-mediated proteolytic process that is presumably regulated by a molecular mechanism since the *N*-terminus of AMPD shares with calpastatin a regulatory domain that might exert a protective role against the fragmentation-induced activation of AMPD [55].

## 4. Localization, Site of Biosynthesis and Primary Structure of Rabbit Skeletal Muscle HPRG

HPRG is an approximately 70 kDa glycoprotein that was first isolated by Heimburger *et al.* in 1972 as a human serum protein with high affinity for CM-cellulose [56,57]. Since then, HPRG has been isolated from the plasma of several mammalian species such as rabbit [27], mouse [58], hog [59], and cow [60]. The first amino acid sequence of HPRG that has been determined, which is the sequence of human HPRG derived from the cDNA sequence [61], was followed by the primary structure of rabbit HPRG [62].

Plasma is the major pool of HPRG, but it is also found in infant urine, colostrums, milk, platelets, megakaryocytes and immuno cells such as monocytes and macrophages [58,63,64]. The tissue distribution of mouse HPRG mRNA, investigated by Northern blot analysis performed on total RNA from liver, spleen, thymus, heart, lung, kidney, brain and testis, demonstrated that HPRG mRNA is produced only in the liver [65]. The same authors reported that RT-PCR analysis failed to detect any HPRG mRNA in immune tissues, suggesting that the HPRG found in immune cells must be acquired from plasma.

Although the physiological role of plasma HPRG remains unclear, it has been implicated in a number of processes, including blood coagulation and fibrinolysis, immune response and transport of metal ions [26]. HPRG has been shown to exhibit an angiogenesis function since HPRG was found to inhibit the antiangiogenic effect of TSP-1 [66]. In contrast, the His-rich domain within HPRG can interact with cell-surface HS to exert an antiangiogenic effect [67]. Zinc-dependent binding of the His-rich domain of HPRG to HS on endothelial cells is required for the inhibition of angiogenesis [67].

An HPRG-like protein was found in rabbit skeletal muscle AMPD preparation [7]. The *N*-terminal sequence analysis of several tryptic peptides of this protein revealed a striking similarity to the fragments from rabbit plasma HPRG; this suggested the presence of an isoform of HPRG in skeletal muscle [7]. In healthy human skeletal muscle, an anti-HPRG antibody selectively bound to type IIB fibers that are well known to contain the highest level of AMPD activity, suggesting an association of the HPRG-like protein to the enzyme isoform M [8]. An immunohistochemical study performed on human skeletal muscle biopsies from patients with AMPD deficiency and carried out utilising both the anti-HPRG antibody and an anti-AMPD antibody specific for the isoform M demonstrated a correlation between the muscle content of the HPRG-like protein and the level of AMPD activity [11].

It has been shown by Borza *et al.* [62] that the overall architecture of rabbit plasma HPRG is similar to that described for the human protein [61]: the *N*-terminal half of the molecule contains two modules homologous to cystatin (1–112, 113–229), while the *C*-terminal half consists of a histidine-proline rich pentapeptide (H/P-H/P-P-H-G) repeat region sandwiched between two proline-rich regions and a *C*-terminal domain. However, the alignment of rabbit HPRG with the human protein showed that a high homology occurs only at the amino and carboxyl termini. The lower homology observed over the histidine-proline rich region was apparently due to the substitution of histidine residues for proline in the rabbit protein [62]. More recently, the nucleotide sequence of cDNA and amino acid sequence of HPRG from other mammalian species have been deposited in GenBank under the following accession numbers: bovine, AB055894; rat, AF194029; rat1, AB055895 and rat2, AB055896; mouse, AF19028 and AB055897. Recent genome projects have also revealed the complete genome sequences of several mammalian species such as bovine, pig, mouse, rat, cat, dog and rabbit which makes it possible to predict the primary structure of HRG from these species (Accession No. NP_776344, NP_001231568, NP_444406, NP_596919, XP_003991881, XP_005639866 and XP_002716393, respectively). All HPRG from the different mammalian species share a well conserved *C*-terminal domain and two well conserved domains at the *N*-terminus (N1 and N2) that are predicted to possess a cystatin-like fold based on their sequence homology to cystatin proteins [68]. Quite recently, the 1.93 Å X-ray crystal structure of the N2 domain of HPRG purified from rabbit serum has been presented that confirms that the N2 domain has a cystatin-like structure that is composed of a five-stranded anti-parallel β-sheet, twisted around a five-turn α-helix [69].

**Figure 2:**
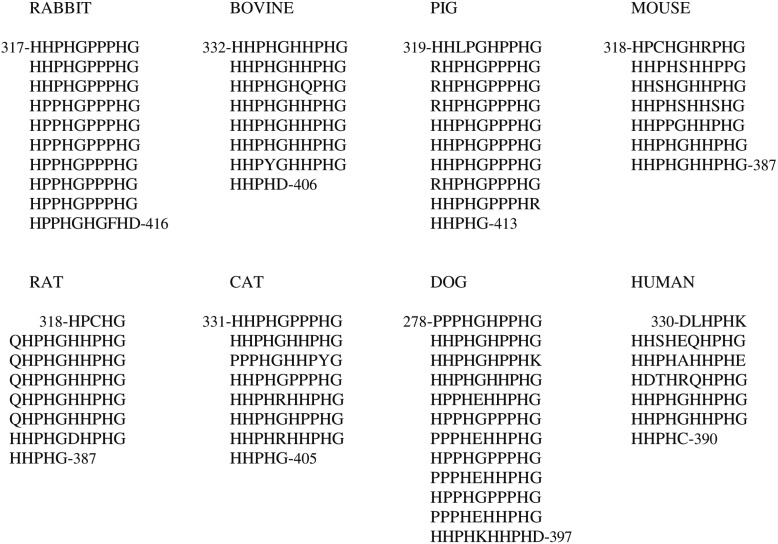
Tandem repeats of consensus decapeptide sequence found in the His-Pro-rich region of Histidine-Proline-Rich Glycoprotein (HPRG) from several mammalian species.

In contrast, the length of the His-Pro-rich region varies among the species and may be better represented by a varying number of tandem repeats of a consensus sequence decapeptide (Figure 2); the length of the flanking Pro-rich region 1 is consequently affected. The histidine residue content retained by the His-Pro-rich region varies in HPRG from the different species from a minimum of 32 in human to a maximum of 51 in dog.

It should be noted that the tandem repeats of the consensus sequence pentapeptide first reported by Borza *et al.* [62] for rabbit plasma HPRG substantially differ from those predicted from the rabbit genome sequence: in contrast with the His-Pro-rich region of the protein of all other mammalian species, the sequence of the rabbit protein reported by Borza *et al.* [62] showed an interruption of the regular repeats that was presumed to be due to a deletion within the rabbit gene. In light of the rabbit genome sequence data, it may therefore be concluded that the primary structure of rabbit plasma HPRG reported by Borza *et al.* [62] is incorrect and that their conclusions concerning the pattern of tandem repeats cannot be substantiated.

**Figure 3:**
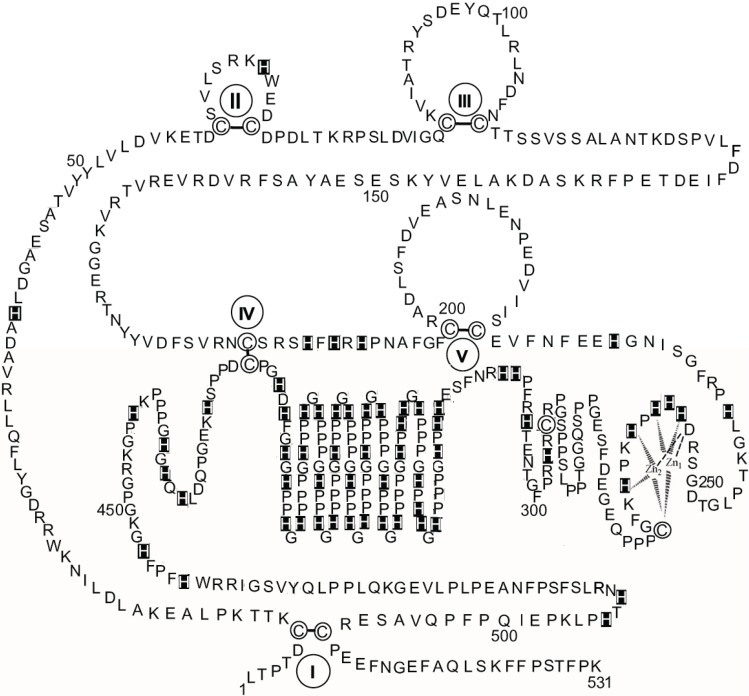
The primary structure of rabbit HPRG. The disulphide bonds are indicated by Roman numerals. His residues are marked. Proposed interactions are shown between two Zn ions and the amino acids localised between residues 253 and 265 in the putative specific dinuclear zinc binding site.

**Figure 4:**
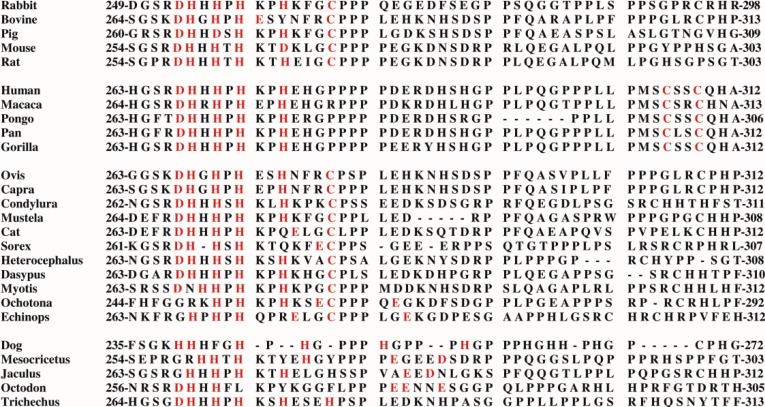
Comparison of the primary structure of HPRG from several mammalian species in the region localized between the *N*-terminal cystatin-like domains and the His-Pro-rich region. The marked amino acid residues are likely to be involved in the spatial formation of a specific zinc binding site.

Figure 3 shows a schematic diagram of the primary structure of rabbit HPRG as predicted by the genome sequence in which the pattern of five disulphide bridges (I–V) connecting Cys-6 to Cys-510, Cys-60 to Cys-71, Cys-87 to Cys-108, Cys-185 to Cys-420, and Cys-200 to Cys-223 is indicated on the basis of the homology to bovine HRG in which the determination of the disulphide bridge arrangement has shown that all the 12 half-cysteine residues found in the protein are involved in the formation of six disulphide bridges [70]. It should be noted that all 12 cysteine residues also found in rabbit HPRG are conserved between the two species suggesting that a sixth disulphide bridge could be inferred in rabbit HPRG, namely between Cys-265 and Cys-295. However, alignment of the amino acid sequence of rabbit and bovine plasma HPRG with the available predicted amino acid sequences of pig, mouse, rat and human HPRG indicates that only 10 of the 12 cysteine residues are totally conserved in all species, suggesting that the five disulphide bridges described above are likely to be essential for the proper folding of the protein of each species. It may be significant that the two cysteine residues that in bovine and rabbit HPRG may be supposed to form one disulphide bond within one of the two proline-rich regions that flank the hystidine-rich region are not conserved in the human protein (Cys-265 of the rabbit HPRG sequence is replaced by proline) nor in the mouse, rat and pig HPRG, where Cys-295 is replaced by other amino acids (Figure 4). This analysis suggests that Cys-265 of rabbit HPRG is not involved in disulphide bond formation and may instead be used by the protein as zinc ligand together with three conserved amino acid residues (namely Asp-253, His-254 and His-258) localised within this region of HPRG (Figure 4), in accordance with the EXAFS analysis of a 2:1 Zn-HPRG complex (see next paragraph) that indicates that zinc is bound to the protein in a dinuclear cluster where two Zn^2+^ ions are bound to histidine residues and one heavier ligand, like carboxylates or cysteine thiolate sulfur [10]. In this view, the *C*-terminal half of rabbit HPRG (Figure 3) consists of a specific zinc binding site (253–265), a histidine-proline rich decapeptide repeat region (317–417) sandwiched between two proline-rich regions (266–299 and 419–455, containing 11 and 10 Pro, respectively) and a *C*-terminal domain (456–531).

As far as the primary structure of HPRG associated to rabbit skeletal muscle AMPD is concerned, it can be assumed that it is most probably superimposable to that represented in Figure 3 since sequencing of the peptides obtained by limited tryptic digestion of several preparations of rabbit muscle HPRG showed identical *N*-terminal sequences (1–7, 8–15, 16–22, 26–34, 35–47, 50–56, 89–93, 94–99, 128–137, 143–148, 177–183, 244–251, 297–305, 461–466, 473–489) to the corresponding fragments of the primary structure of the protein which was recently derived from the rabbit genome sequence.

It should be noted that at the time when the first sequence results obtained for the HPRG fragments liberated by trypsinization of a rabbit skeletal muscle AMPD preparation were published, a clear degree of divergence in the region containing residues 485–487 was observed between the sequence of rabbit plasma HPRG available at that time (Q-L-L) [62] and that of the novel component of the enzyme (S-F-X) [7], the last one being in agreement with the primary structure of rabbit HPRG that has been recently predicted from the rabbit genome sequence (S-F-S). The possibility that the primary structure of rabbit plasma HPRG reported by Borza *et al.* [62] was wrongly determined is further strengthened by the presence in the sequence reported by Borza *et al.* [62] of one methionine residue (Met-424); in contrast, no methionine residues are present in the HPRG sequence that has been derived from the rabbit genome sequence. It has recently been observed [71] that none of the three constituent peptides of the incubation mixture at the end of the CNBr treatment of HPRG dissociated from rabbit skeletal muscle AMP deaminase showed the amino-terminal sequence corresponding to the sequence G-P-P-P following Met-424 in the primary structure submitted by Borza *et al.* [62] for rabbit plasma HPRG. On the contrary, after separation by SDS/PAGE of the three components, electroblotting and sequencing of the slowest migrating 75 kDa band revealed a single sequence corresponding to the uncleaved protein, having L-T-P-T-D-C-K-T-T-K-P-L-A-E as *N*-terminus whilst the *N*-terminal sequences detected for the other two peptides (H-G-P-X-D and (V-I-G-Q-X) were clearly derived from the cleavage of peptide bonds at the *C*-terminal side of Asp since they corresponded to the HPRG fragments yielded through cleavage of the 416-Asp-His and 82-Asp-Val bonds and having H-G-P-C-D and V-I-G-Q-C as *N*-terminus, respectively; the “X” in both sequences is totally compatible with the genome-derived sequence as C cannot be detected easily by conventional Edman degradation. These results indicate that there are no methionines present in skeletal muscle HPRG as well as in plasma HPRG and that the fragments obtained by CNBr treatment of the protein were produced from cleavage in 70% formic acid of peptide bonds involving the *C*-terminal side of aspartyl residues whose acid lability is well known [72,73].

Altogether, the above observations are in agreement with the results of a recent investigation on the origin of skeletal muscle HPRG that have reported that skeletal muscle cells do not synthesize the protein but can instead actively internalize plasma HPRG [74] and strongly suggest that the HPRG found in skeletal muscle is identifiable with the protein that must be acquired from plasma.

## 5. Characterization of the Zinc Binding Site of Rabbit Skeletal Muscle HPRG

The histidine-rich domain of HPRG has been proposed to mediate interactions with transition metals although no evidence of a specific binding was given. It has been established that HPRG from rabbit serum binds Hg^2+^, Cu^2+^, Zn^2+^, Ni^2+^, Cd^2+^, Co^2+^ in descending order of binding affinity [75]. The effects of metals on the fluorescence of the protein [27], showed that HPRG interacts with various divalent metal ions with an apparent stoichiometry of 10 (Cu^2+^, Co^2+^, Ni^2+^ and Hg^2+^) to 20 (Zn^2+^ and Cd^2+^) metal ions bound per HPRG molecule. The existence of some non-overlapping sites was suggested by the sigmoidal binding of Zn^2+^ and Cd^2+^ (Hill coefficient, *h* = 4.4 and 2.8, respectively) while the other metals were bound in a non-cooperative manner. Moreover, only Zn^2+^ and Cd^2+^ caused an enhancement rather than a quenching of fluorescence [27]. Altogether, these results were interpreted as an indication that all the metal ions probably share the same set of binding sites although the interaction of each metal with the protein may vary because of the involvement of a different group of protein ligands in a different way. Nevertheless, the binding of zinc is of particular interest since it has been reported to be essential for the interactions of HPRG with heparin and endothelial cell heparan sulfate [76,77,78] and of the His-Pro-rich domain of HPRG with plasminogen [79,80] and cell surface tropomyosin [81]. It should be noted that HPRG has probably no role in the chelation of zinc in the blood, since the vast majority of this metal present in human blood 20 µM) is bound to serum albumin that has a concentration (250 µM) much higher than that of HPRG (2.5 µM) [75]; if the relative rate of uptake or release of zinc from serum albumin and HPRG can be determined and compared, a role for HPRG in the exchange of zinc or modulating the intracellular availability of zinc might be predicted.

The interaction of the HPRG component of rabbit skeletal muscle AMPD protein with metals was assessed directly by monitoring changes in UV-absorption [10]. When compared with HPRG alone, the protein in the presence of increasing equivalents of Zn^2+^ or Cu^2+^ or Ni^2+^ showed an increase in absorption around 275 nm that reached saturation with a binding stoichiometry of near 50 metal ions per HPRG molecule. Titration of the interaction of HPRG with Zn^2+^ or Ni^2+^ showed a sigmoidal relationship (*h* = 4.0 and 3.5, respectively) indicating that these metals are bound in a cooperative manner with interactions among various sites. Cu^2+^ bound to HPRG with the same apparent stoichiometry observed with Zn^2+^ and Ni^2+^ but showed a minor positive cooperativity of binding (*h* = 1.5). These results are in agreement with the reported effects of metals on the fluorescence of rabbit plasma HPRG [27] and demonstrate that most of such a large number of metal binding sites are non-specific and that most of the zinc is simply adventitiously bound.

In order to minimize the effect of a distribution of zinc binding sites and to enhance the chance of characterizing the structure of a specific binding site, an investigation by X-ray absorption spectroscopy (XAS) of the zinc binding behaviour of rabbit skeletal muscle HPRG that had been isolated from the AMPD complex was carried out with a sample obtained by adding two equivalents of zinc to the protein [10]. Inspection of the XAS data obtained with HPRG samples containing eight or 15 equivalents of zinc showed that different EXAFS patterns were superimposed on the spectrum obtained with the 2:1 Zn-HPRG complex. The EXAFS analysis of the 2:1 Zn-HPRG complex showed that zinc is bound to the protein, most probably in a dinuclear cluster where each Zn^2+^ ion is coordinated, on average, by three histidine and one heavier ligand, most likely a sulfur from a cysteine. In principle, the EXAFS analysis gives only the average zinc coordination in the sample and does not establish whether one zinc ion is in a histidine-only environment (*i.e.*, Zn-N(His)_4_) and the other in a N/S coordination (*i.e.*, Zn-N(His)_2_,S_2_), or if the zinc binding site is a more symmetric one with common bridging ligands. However, the evidence from the EXAFS data suggested that the zinc binding site in HPRG may host two metal ions at a distance of about 3.7 Å which is typical of dinuclear first transition-row metal sites with bridging ligands like carboxylates or cysteine thiolate sulfur. Taking into account the EXAFS results, four amino acid residues (namely Asp-253, His-254, His-258 and Cys-265 of rabbit HPRG) that are conserved in almost all mammalian HPRG which have been sequenced and two additional aminoacidic ligands that might be His, Asp or Glu residues (namely His-256 and His-261 of rabbit HPRG) are candidates to be in contact with zinc in a specific dinuclear binding site (253–265 region of rabbit HPRG, Figure 3). It should be noted that this view is challenged by the observation that in human plasma HPRG Cys-265 of the rabbit protein sequence is replaced by proline. However, in the proline-rich region that flanks that of the putative zinc binding site of other mammalian species, human plasma HPRG distinctively presents the sequence MSCSSC (residues 304–309, see Figure 4) that contains the MxCxxC motif, conserved in many metal transporters [82]. It has been shown that in yeast the chaperone Atx1, which shuttles Cu to an intracellular Cu transporter, contains a conserved MxCxxC sequence motif that binds Cu^+^ with two cysteines [83,84,85]. Homologues of Atx1 have been identified in humans, bacteria, plants and animals [86,87]. The amino acid sequence MxCxxC is also conserved in many soft-metal transporters that are involved in the control of intracellular concentration of other metal ions such as Hg(II), Zn(II), Cd(II) and Pb(II). In particular, ZntA, a Zn-trafficking P-type ATPase, binds ZnII) via an aspartate O atom in addition to the two cysteine S atoms and it has been suggested that the presence of aspartate could enhance the binding specificity for zinc ions [88]. The *N*-terminal domain of CCS, the copper chaperone for superoxide dismutase (SOD1), harbors the same MxCxxC site that in the case of Atx1 serves a two-fold purpose in capturing the metal and in direct metal transfer to its target [89], whilst in CCS it only ensures the recruiting of the metal. The carboxyl-terminal domain of CCS contains a copper binding CXC motif critical to the metal transfer activity of the protein [90]. The above observations suggest that in human HPRG a zinc binding site is present where the metal is coordinated by Asp-267, His-268, His-272, that are conserved in mammalian species, and/or by one or two Cys from the MSCSSC region (residues 304–309). This model can be extended to all primates, since all the primary structures of HPRG predicted from the complete genome sequences of gorilla (XP_004038233), pan (XP_001154095), pongo (XP_002814431) and macaca (XP_001090791) show the presence of the MSCSSC sequence motif (Figure 4). Interestingly, in the proline-rich region that follows the His-Pro-rich domain of human HPRG four cysteine residues are present (Cys-390, Cys-409, Cys-410, Cys-434) that are well conserved in the Primates (with the exception of macaca HPRG that shows the sequence CHHC instead of the sequence HCCH that is shared by the other Primates and includes Cys-409 and Cys-410 of the human protein) but are absent in HPRG of rabbit, rat, mouse and pig.

Altogether, the above considerations strongly suggest that a specific zinc binding site exists in mammalian HPRG that is harbored by a domain, distinct from the His-Pro-rich region, that is probably shared by the different species of mammal although the interaction of zinc with the protein from these different species may vary because of the involvement of a different group of protein ligands. This hypothesis is strengthened by the observation obtained when the comparison of the primary structure of the region of the putative zinc binding site is extended to HPRG from several other species of different orders of mammal (Figure 4).

It can be seen that whilst in the HPRG from Ovis (XP_004003109), Capra (XP_005675216), Condylura (XP_004675463), Mustela (XP_004787528), Cat (XP_003991881), Sorex (XP_004603311), Heterocephalus (XP_004834882), Dasypus (XP_004473765), Myotis (ELK31606), Ochotona (XP_004591376), Echinops (XP_004713523) the potential zinc ligands are similar to those of rabbit HPRG, the primary structures of the protein from Dog (XP_005639866), Mesocricetus (XP_005071757), Jaculus (XP_004654353), Octodon (XP_004640977), Trichechus (XP_004382388) show that Cys-265 of the rabbit protein sequence is replaced by other amino acids, indicating that a histidine-only binding site (such as in dog) or an arrangement composed of His residues and carboxylate moieties from Asp and/or Glu residues might represent other ways zinc may be transferred to its target.

**Figure 5:**
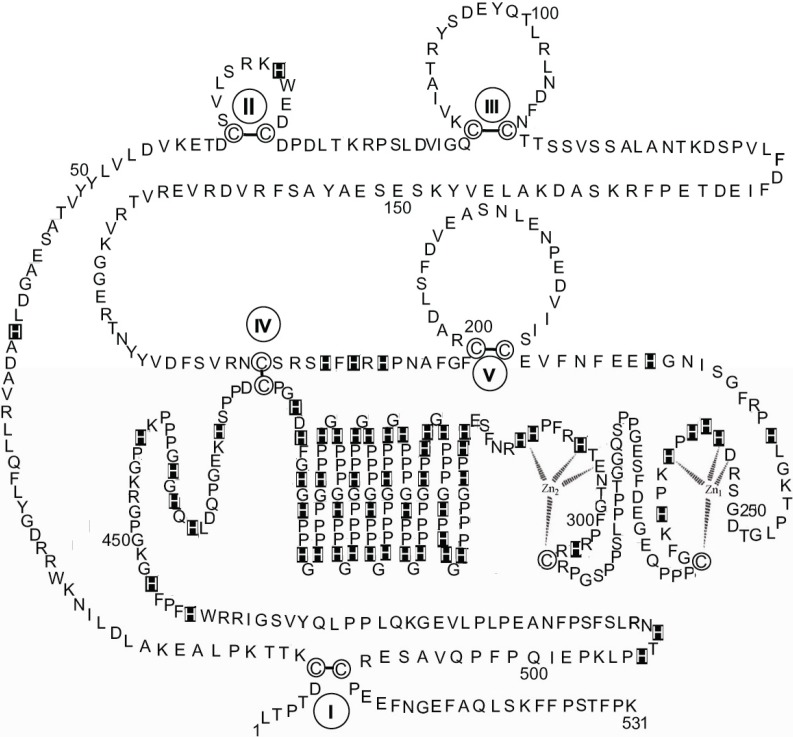
Hypothesis of a specific dinuclear zinc binding site in rabbit HPRG involving both Cys-265 and Cys-295. Proposed interactions are shown between two Zn ions and the amino acids localised in the 253–311 region.

Whilst the above considerations based on the comparative sequence data lead to speculative conjectures, a sounder result is reached when the attempt is made to fit the dinuclear zinc binding site model that has been described for HPRG dissociated from rabbit muscle AMPD in the 253–311 residue region of rabbit HPRG that includes the Pro-rich region 1, thus involving both Cys-265 and Cys-295 in the structure of the zinc binding site. Figure 5 shows that two possible 4-coordinate zinc sites are found, in both of which the ligands might be one cysteine thiolate and three among carboxylates of aspartate or glutamate and histidine imidazoles. In the case of two 5-coordinate zinc ions (not shown) a water/hydroxide molecule would bridge between them.

## 6. Participation of HPRG in the Assembly and Maintenance of Skeletal Muscle AMPD by Acting as Zinc Metallo Chaperone

By coupling the finding that rabbit muscle HPRG as well as the catalytic subunit of AMPD is able to bind zinc in a dinuclear metal binding site with the previous suggestion of the presence in the whole AMP deaminase of additional zinc not required for activity [32] and by considering the absence of significant differences in the kinetics of AMPD with different HPRG content [10], the addition of HPRG into the family of metallochaperones has been envisaged, suggesting that HPRG may enhance the stability of AMPD *in vivo* through insertion of zinc or by modulating the intracellular zinc availability [10]. This view is strengthened by the results of an investigation of the effects of the isolation by zinc-affinity chromatography of the HPRG component of rabbit skeletal muscle AMPD [9]. When the whole enzyme was loaded on a Zn^2+_^charged affinity column under denaturing and reducing conditions, only the HPRG component was specifically retained on the column while the catalytic subunit emerged in the void volume and precipitated immediately in the flow-through. The protein precipitation has been ascribed to a disruption of the association between the two components of the enzyme due to the selective binding of HPRG to the resin. HPRG was only eluted with an EDTA-containing buffer that strips Zn^2+^ from the gel [9]. The observation that separation of HPRG induces a marked reduction in the solubility of the catalytic subunit of skeletal muscle AMPD strongly suggests a role of HPRG in the maintenance of the native quaternary structure of the enzyme that could be envisaged from the formation of a 1:1 HPRG-AMPD molecular adduct [9]. Further evidence that supports this conclusion is the aggregation observed with the cloned AMPD1 subunit on gel filtration, where the absence of the HPRG subunit results in aggregation of the cloned protein [42].

The HPRG that was isolated by zinc-affinity chromatography was homogeneous showing an apparent 95 kDa MW and the *N*-terminal sequence L-T-P-T-D-X-K-T-T-K-P-L-A-E-K-A-L-D-L-I, corresponding to that of rabbit plasma HPRG. The incubation with peptide-N-glycosidase F promoted the reduction of the apparent MW of isolated HPRG to 70 kDa, characterizing it as a N-glycosylated protein [9].

As described in the previous paragraph on the characterization of rabbit skeletal muscle AMPD as a metalloenzyme with a dinuclear zinc site, tryptic digests of different enzyme preparations demonstrated the existence of two different protein conformations that are dependent on the presence/absence of a zinc ion connecting the *N*-terminal and *C*-terminal regions of AMPD. Comparison of the HPRG fragments that are produced during the two qualitatively different limited tryptic digestions of fresh preparations of rabbit skeletal muscle AMPD (Table 1) reveals that when the extent of digestion of AMPD is apparently limited to the *N*-terminal region residues (1–95) and to the central region residues (176–467) of the enzyme (Table 1A), there is also evidence of a digestion at the HPRG *N*-terminus (residues 1–34, 94–99), in two central regions of the protein (residues 244–251, 297–305) that encompass the putative zinc specific binding site (residues 253–265, see Figure 3, or residues 253–311, see Figure 5) and in the region (residues 461–489) that follows the Pro-rich region 2. In contrast, when the digestion of AMPD affects the binding region of the Zn1 ion at the *C*-terminus (residues 609–654) (Table 1B) whilst the fragmentation of the *N*-terminus of the enzyme is limited to the 1–52 region, profound changes also within the HPRG structure ensue as revealed by the observed extension of the fragmentation of the *N*-terminal region to both cystatin modules (residues 1–183) whilst the digestion in the central region liberates only the 473–489 fragment. The qualitative differences observed between the products of the two different limited digestions demonstrate a differing susceptibility of each separate domain of AMPD and HPRG to cleavage by trypsin, and therefore reveal the existence of two distinct but linked protein conformations of the two components of the enzyme. This is consistent both with the presence of specific interactions between the *N*-terminal and *C*-terminal regions of rabbit skeletal muscle AMPD as suggested by the structural role that can be played by zinc in stabilizing one among the possible enzyme conformations and with the presence in the HPRG component of a disulfide bridge connecting the *N*-terminal and *C*-terminal regions (Figure 3). The observation that the tryptic peptides derived from the *N*-terminal region of HPRG are liberated in constant association with fragments derived from the AMPD *N*-terminus suggests that the *N*-terminus of HPRG should be exposed as well as the AMPD region that encompasses the regulatory domain associated with the peculiar inhibition of the enzyme by ATP [41,50,51] and strengthens the hypothesis of a physiological role of HPRG in preserving the molecular integrity of the enzyme. The protective role of HPRG against the fragmentation of AMPD by thiol proteases is suggested by the presence of two modules homologous to cystatin (1–112, 113–229) at the *N*-terminus of the protein (Figure 3) and by the observation that the rate of the fragmentation of HPRG-enriched AMPD on storage is reduced [10].

**Table 1:** *N*-terminal sequences of peptides that are produced by limited tryptic digestion of different fresh preparations of rabbit skeletal muscle AMPD. Numbers indicate the collocation of the peptides in the sequences derived from rabbit genomic sequences of skeletal muscle AMPD (isoform M) and HPRG (accession numbers XM_002715794 and XP_002716393, respectively). The peptides were subjected to *N*-terminal analysis after their isolation by HPLC of the soluble fraction obtained after acid precipitation of the digests obtained by trypsinization of different preparations of AMPD (60 min at pH 7.0, trypsin/AMPD 1:80, *w*/*w*). (**A**) *N*-terminal sequences of the fragments of AMPD and HPRG liberated when the digestion of AMPD was limited to the *N*-terminal region (residues 1–95) and to the central region (residues 176–467); (**B**) *N*-terminal sequences of the fragments of AMPD and HPRG liberated when the digestion of AMPD affected a less extended *N*-terminal region (1–52), the central region (residues 176–483) and the binding region of the Zn_1_ ion at the *C*-terminus (residues 632–654).

A		B	
AMPD	HPRG	AMPD	HPRG
4–11	16–22	4–11	8–15
6–11	26–34	6–11	16–22
12–18	94–99	12–18	26–34
19–23	244–251	19–23	35–47
24–30	297–305	24–30	50–56
36–55	461–466	36–41	89–93
58–72	473–489	42–52	128–137
76–79		175–189	143–148
80–92		190–196	177–183
81–95		217–230	473–489
176–184		456–461	
322–329		462–467	
419–425		470–477	
462–467		478–483	
		609–617	
		632–640	
		641–654	

As far as the functional role of HPRG in the AMPD-HPRG complex is concerned, the hypothesis that the HPRG component behaves as a zinc metallochaperone deserves further consideration. Several findings about the AMPD-HPRG complex parallel the well documented association of superoxide dismutase (SOD1) with its copper chaperone (CCS) for which also a crystal structure exists [91]. The *N*-terminal domain of CCS contains the MxCxxC site that ensures recruitment of the metal. The carboxyl-terminal domain of CCS contains a copper binding CXC motif critical to the metal transfer to a histidine-only site in SOD1 by a mechanism which most probably involves the formation of an intermolecular disulfide bridge [91]. The presence in the HPRG molecule dissociated from rabbit skeletal muscle AMPD of a dinuclear cysteine-bridged zinc binding site [10] allows one to envisage the possibility that the novel component of AMPD could behave in a fashion similar to CCS in enhancing the stability of the metalloenzyme through insertion of zinc. It should be noted that in mammalian HPRG, capturing the metal is most probably accomplished by the His-Pro-rich region whilst the zinc transfer to its target AMP deaminase should involve the zinc specific binding site that is variously represented in different orders of mammalian (see Figure 4). Primate HPRG harbors the same MxCxxC site that in CCS ensures the recruiting of the metal. However, the MxCxxC site may serve different purposes as it has been shown in the case of the chaperone Atx1 where it is also involved in direct metal transfer to its target [89].

## 7. Conclusions

A model has been proposed for the dinuclear Zn site in rabbit skeletal muscle AMPD (Figure 1A) and a model has been presented for the interactions of the substrate at the dinuclear cocatalytic Zn site of the enzyme (Figure 1B). The two Zn ions in the AMPD metallocenter operate together as a catalytic unit, but play different roles in the catalytic mechanism; Zn_1_ acts to polarize the nucleophile water molecule, whilst Zn_2_, possibly in co-operation with Zn_1_, acts transiently as the receptor for an activating substrate molecule (Figure 1B). The ligand displacement that presumably occurs when substrate enters the zinc coordination sphere of AMPD is consistent with a *cocatalytic* mechanism. We suggest that a specific zinc binding site exists in mammalian HPRG that is harbored by a domain that is distinct from the His-Pro-rich region. We also demonstrate the participation of HPRG in the assembly and maintenance of skeletal muscle AMPD by acting as zinc chaperone.

Further studies are necessary to identify the domains of HPRG that are required for contact with AMPD and whether a mechanism exists to transfer zinc between the two proteins. However, the data is consistent with zinc not being required for HPRG/AMPD interaction since the spectrum of the Zn-AMPD-HPRG complexes with various molar ratios of HPRG did not show any evidence of sulfur coordination to zinc, thus demonstrating that no zinc is bound to HPRG when the ternary complex of HPRG with Zn and the AMPD catalytic subunit is formed.
